# *Lactobacillus* Adhesion to Mucus

**DOI:** 10.3390/nu3050613

**Published:** 2011-05-20

**Authors:** Maxwell L. Van Tassell, Michael J. Miller

**Affiliations:** 1 Department of Food Science and Human Nutrition, University of Illinois, 905 S. Goodwin Ave., Urbana, IL 61801, USA; Email: vantass2@illinois.edu; 2 Division of Nutritional Sciences, University of Illinois, 905 S. Goodwin Ave., Urbana, IL 61801, USA

**Keywords:** adhesion, binding, mucin, mucus, MUC2, lactobacillus, MucBP, probiotics

## Abstract

Mucus provides protective functions in the gastrointestinal tract and plays an important role in the adhesion of microorganisms to host surfaces. Mucin glycoproteins polymerize, forming a framework to which certain microbial populations can adhere, including probiotic *Lactobacillus* species. Numerous mechanisms for adhesion to mucus have been discovered in lactobacilli, including partially characterized mucus binding proteins. These mechanisms vary in importance with the *in vitro* models studied, which could significantly affect the perceived probiotic potential of the organisms. Understanding the nature of mucus-microbe interactions could be the key to elucidating the mechanisms of probiotic adhesion within the host.

## 1. Introduction

Lactobacilli are of significant importance to food industries due to their involvement in the production of various fermented dairy, meat, and vegetable foods. Also important to the health industry, lactobacilli are used as probiotics due to their health-promoting effects when consumed. One of the frequently exploited activities used to screen probiotic candidates is adhesion to the host gut, which is presumed to be requisite for sufficient host-interaction to confer health benefits [1]. Various *in vitro* models are used for the study of bacterial adhesion because of the complexity of studying the *in vivo* system. However, these models are simplifications of *in vivo* situations, resulting in limited conclusions. One significant aspect of the gastrointestinal (GI) tract that is easy to overlook when studying bacterial adhesion, and choosing models with which to measure adhesion, is the presence of a mucus layer between the lumen and GI epithelial cells. The presence of mucus is particularly relevant in the colon, where mucus is thickest and microorganisms are most abundant.

The layer of mucus bound to GI epithelia is formed from a continuous gel matrix composed primarily of complex glycoproteins that acts as a barrier to protect the host from harmful antigens and promote luminal motility. This layer of mucus is the first physical barrier to host-cell stimulation by bacteria in the gut. Adhesion to this mucus is therefore the first step required for probiotic organisms to interact with host cells and elicit any particular response. In the human intestinal tract, the layer of mucus may vary in thickness from about 30 to 300 µm, generally increasing in thickness from the small intestine to the rectum, but the layer of mucus most closely bound to the epithelial layer rarely contains any bacteria at all [2,3]. 

Numerous studies have characterized interactions between bacteria and host epithelia that induce alterations in host mucosal response [4,5,6] but how changes in mucus composition affect adhesion by gut microorganisms is not well understood. Likewise, exposure to mucus during growth has been shown to affect bacterial gene expression [7], but resulting changes to adhesion are not well recognized. Additionally, existing studies for bacterial adhesion show great variability due to a lack of standardization, complicating the interpretation of data from the current literature [8].

In this review, we will examine the composition of the mucus layers protecting GI epithelial tissue, which is considered to be the primary location of host-probiotic interaction [9]. Our focus will be on its relevance to *Lactobacillus* species, commensal bacteria of the human gut that are used extensively in commercial probiotic supplements and contain the most widely studied probiotic species in scientific literature. Our goal is to provide a framework for a better understanding of the role that mucus plays in probiotic-host interactions.

## 2. Intestinal Mucus

The epithelial tissue that forms the lining of the intestine is composed of various columnar cell types. Scattered across the length of the intestine, and all mucosal tissues, are goblet cells. These cells are unicellular glands that produce glycoproteins called mucins, which give mucus its characteristic viscoelastic physical properties. Secreted mucins polymerize to form the matrix that provides the structural foundation of the mucus layer resulting in protection from pathogens, enzymes, toxins, dehydration and abrasion [10]. Goblet cells produce secretory mucin at a basal constitutive level under normal physiological conditions to maintain this protective layer of mucus, which is exposed to the harsh luminal environment and constantly eroded by luminal particulates and intestinal peristalsis [11]. 

Table 1 shows a reported 21 *MUC* genes code for the protein cores of mucins in humans. Gastrointestinal mucins are either translocated to the membrane surface or secreted into the mucous gel. Mucins are also either neutral or acidic, depending on their glycosyl modification. These categories can be further subdivided to account for greater variation in mucin structure [12]. 

**Table 1:** Known human *MUC* genes, their functions and locations.

Gene	Organisms with known homologues ^1^	Function ^2^	GeneAtlas location of highest expression ^2^	Type	Selected references
*MUC1*	Dog, cow, mouse, rat, rabbit	Cellular signal transduction, barrier activity	Lungs	Membrane	[13,14]
*MUC2*	Chimpanzee, dog, chicken	Primary extracellular matrix constituent in colon, lubricant activity	Colon	Secretory	[14,15]
*MUC3A*	Rat, mouse	Involved in epithelial cell protection, adhesion modulation, and signaling	Various	Membrane	[16]
*MUC3B*	Rat, mouse	Unknown, possibly cellular signal transduction	Various	Membrane	[16]
*MUC4*	Many mammals, chicken, frog, platypus	Involved in intestinal epithelial cell differentiation, renewal, lubrication	Colon	Membrane	[17,18]
*MUC5B (MUC9)*	Chimpanzee, zebrafish, mouse, chicken, more	Unknown, primarily lubricant	Various	Secretory	[19,20]
*MUC5AC*	Chimpanzee, rat, zebrafish	Major component of airway mucus involved in intestinal epithelial cell differentiation	Trachea, Lungs	Secretory	[21,22]
*MUC6*	Chimpanzee, dog, mouse, chicken	Unknown, involved in renal morphogenesis processes	Pancreas, digestive and reproductive systems	Secretory	[22,23,24]
*MUC7*	Chimpanzee, cow, rat	Facilitating the clearance of oral bacteria	Salivary Gland	Secretory	[25,26]
*MUC8*	Unknown	Unknown	Trachea	Secretory	[27]
*MUC12 (MUC11)*	Cow, M.grisea, *N. crassa*, rice	May be involved in epithelial cell regulation	Colon	Membrane	[28]
*MUC13*	Chimpanzee, dog, mouse, rat	Barrier function in epithelial tissues	Pancreas, small intestine, colon	Membrane	[29]
*EMCN (MUC14)*	Dog, cow, mouse, rat, chicken	Interferes with the assembly of focal adhesion complexes	Fetal lung, uterus, thyroid	Membrane	[30]
*MUC15*	Chimpanzee, cow, mouse, rat	Barrier function in epithelial tissues	Testis leydig cell	Membrane	[31]
*MUC16 (CA125)*	Chimpanzee, dog, mouse, chicken	Unknown, plays a role in ovarian cancer	Lymph nodes, respiratory tract	Membrane	[32,33]
*MUC17 (MUC3)*	Chimpanzee, *S. pombe*, *S. cerevisiae*, and *K. lactis*	Extracellular matrix constituent, lubricant activity	Small intestine, stomach	Membrane	[34,35]
*MCAM (MUC18, CD146)*	Chimpanzee, dog, mouse, rat, zebrafish	AKA “melanoma cell adhesion molecule”, cell-cell adhesion	Various	Membrane	[36,37]
*MUC19*	Chimpanzee, dog, mouse, rat, frog	Major gel-forming mucin in the human middle ear	Secretory cells of the ears and eyes	Secretory	[38]
*MUC20*	Chimpanzee, dog, cow, mouse, rat	Cellular signal transduction	Intestine, respiratory and urinary tract	Membrane	[39]
*MUC21*	Chimpanzee, cow, mosquito, and *A. thaliana*	Unknown, mediates cell adhesion	Unknown	Membrane	[40,41]
*CD164 (MUC24)*	Chimpanzee, dog, cow, mouse, rat, chicken, zebrafish	Regulates stem cell localization to the bone marrow	Thyroid, placenta, intestine, immune cells	Membrane	[42]

^1^ Via HomoloGene [43] database; ^2^ via GenAtlas [44] and BioGPS [45] databases.

### Mucin Genes and Modifications

**Figure 1:**
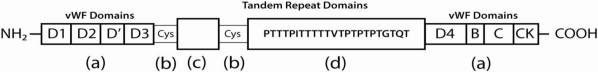
Diagram of the MUC2 protein core. The protein termini contain cysteine-rich regions homologous to von Willebrand Factor (vWF) domains (**a**); The *N*-terminal regions of MUC2 proteins contain vWF domain homologs D1, D2, D′, and D3 and the *C*-terminal regions contain vWF domain homologs D4, B, C, and CK. These terminal domains are responsible for the extensive polymerization between mucin monomers, along with the cysteine rich interruptions between glycosylated tandem repeats (**b**); The first of two repetitive domains (**c**) contains 21 repeats of an irregular motif, whereas the second domain (**d**) is formed of 50-115 tandem 23aa motifs (PTTTPITTTTTVTPTPTPTGTQT). Threonines in the repeats are O-glycosylated, forming a densely packed envelope of short, branched carbohydrate chains surrounding these regions.

Gastrointestinal MUC proteins contain characteristic tandem repeats of threonine, proline, and serine residues, where O-glycosidic linkages occur between the protein core and *N*-acetylgalactosamine (GalNAc) termini of oligosaccharides [46,47]. Neither the amino nor the carboxy termini of secretory mucins are generally glycosylated [48], but contain cysteine rich regions that promote intermolecular disulfide bonding (as shown in Figure 1). The dense glycosylation of the protein core and intermolecular bonding of the terminal regions effectively protects the mucin polymers from protease activity, preserving the protective structural matrix [49].

The predominant genes expressing membrane-bound mucins in human colonic goblet cells are *MUC1*, *MUC3A/B*, *MUC4*, and *MUC12*. Membrane-bound mucins could play a role in immunomodulatory effects of bacterial interactions with the epithelial membrane when the secretory mucin matrix is bypassed [50], however bacteria more frequently come in contact with secretory mucins considering the majority of bacteria only inhabit the outer portions of the mucus layers [51]. *MUC2* is the principal secretory mucin gene expressed in the colon, comprising the majority of the mucous gel protecting the underlying tissue [52]. The role and mechanisms of mucin in innate immunity is reviewed more thoroughly by Dharmani *et al.* [53] and for a more detailed structural analysis of MUC2, see Allen*et al.* [15].

Oligosaccharide chains are affixed to MUC proteins by membrane-bound transferases in the Golgi apparatus and endoplasmic reticulum of goblet cells. GalNAc is affixed to the mucin protein from a sugar-nucleotide donor and a collection of specific glycosyltransferases continues to add residues, resulting in an oligosaccharide with a particular structure and terminus [54,55]. Glycosylation biosynthesis pathways are highly complex; glycosyltransferase gene expression levels, variability in spatiotemporal concentrations of enzymes, cofactors, and substrates, as well as the number of branching configurations possible all contribute to the wide range of potential protein-modifications [55]. This leads to glycoproteins forming from the same mucin gene product that will vary in glycan modification with location or tissue.

The oligosaccharide modifications can comprise up to 80% of the weight of a mucin and vary in length and structure. Secreted colonic mucins commonly contain side-chains of 4-15 monosaccharides with galactose and GalNAc backbones and branched chains terminating with GalNAc, fucose, or sialic (neuraminic) acid to varying degrees [56,57].

The predominance of acidic mucin subtypes, those with side-chains containing terminal ester sulfates and sialic acid groups, varies by location in the GI tract from species to species, as does the type of acidic modification most heavily expressed [58]. The presence of acidic side-chains can result in greater inhibition of bacterial growth *in vitro* [59] and reduced enzymatic degradation [60,61], but what causes the prevalence of these modifications in different parts of GI tissues is likely due to the tissue-dependence of specific collections of glycosyltransferase enzymes [62].

intestinal mucin polymers are considered nutritive glycans for commensal bacteria in the promotion of their residence and associated benefits [63,64]. Host glycosylation patterns in the gut may have coevolved with intestinal microbiota to accommodate the filling of niches beneficial to the host [65,66]. Host provision of mucin oligosaccharides specific to particular bacterial enzymes could provide a nutritional advantage to bacteria with those enzymes and differential expression of mucin oligosaccharides by tissue could hypothetically regulate host-microbe interactions to direct certain microbial populations to fill particular host-niches. So-called host “legislation” of glycosylation to promote particular microbial populations is evaluated in greater depth in a review by Patsos and Corfield [67]. Whether this plays a crucial role in *Lactobacillus* adhesion or is primarily a mechanism of promoting maintenance of other commensal microbiota is currently unclear.

One broad example of host legislation comes from the analysis of mucin oligosaccharide composition along the human intestinal tract, which showed that certain glycosylation patterns were conserved regionally despite inter-individual variation [68]. A gradient of sialylated mucin concentration was observed, decreasing from the ileum to the colon, running against an increasing gradient of more heavily fucosylated mucin.

A more specific example of microbial legislation by hosts lies in the presence of *O*-glycans on mucin that exhibit Lewis type or blood group ABO antigens. The secretor genes that determine host blood type also control the specificity of the ABO blood group type terminal glycosides of certain mucin oligosaccharide chains [69]. The glycosyltransferase responsible for blood group antigen precursors has been identified in secretory tissues producing mucins and glycoproteins [70]. There is evidence that populations of bacteria that produce specific blood type antigen-degrading glycosidases are present at levels 50,000 times greater in individuals with that particular blood type [71]. 

While evidence of mucin oligosaccharide degradation by bacteria is fairly well established [64,72,73,74,75], the dramatic impact of blood type on the composition of enteric microbial populations could imply that there is some degree of binding preference at play with host glycan legislation as well. This is supported by evidence of bacteria binding with human milk oligosaccharides, which can exhibit structural similarities with mucin oligosaccharides and blood type antigens [76]. 

**Figure 2:**
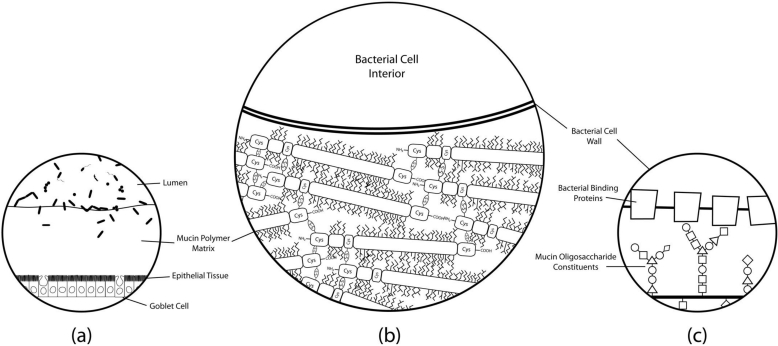
Simplified histological cross-section of microbial adhesion to the colonic mucosal surface at various magnifications. (**a**) *The layer of mucus atop colonic epithelial villi.* Goblet cells can be seen interspersed throughout the columnar enterocytes, producing secretory mucin that makes up the gel matrix. The microbial communities residing in and on top of the mucus layer can only be found at substantial concentrations in the outermost regions of the mucus; (**b**) *The mucus-bacteria interface.* The mucin molecules polymerize to form the mucus layer matrix to which cells adhere. Extensive disulfide bonding between cysteine-rich regions of the mucin protein cores creates the characteristic viscoelastic properties of mucus. Oligosaccharide modifications of mucin protein cores form “bottle-brush” regions providing substrate for adhesion to binding proteins on bacterial cell surfaces; (**c**) *A proposed molecular mechanism of adhesion.* Evidence suggests that putative mucin-binding proteins anchored to the bacterial cell wall may interact with the glycosyl modifications of the mucin proteins to promote adhesion of the cell to the mucus layer. Mucin oligosaccharide structures vary due to tissue and cell-specific glycosyltransferase expression levels, so the specificity of particular oligosaccharide moieties may lead to preferential binding of particular bacteria to different host niches.

Glycosidases of lactic acid bacteria have been fairly well characterized in terms of oligosaccharide breakdown and metabolism [77,78], but knowledge of glycoconjugate adhesion remains poorly described. Figure 2 displays a model of molecular binding mechanisms that may play a role in host-bacteria interactions. Elucidation of these binding mechanisms may be the key to understanding adhesion of lactobacilli in the gut.

For a more detailed characterization of mucin production, structure, and host function see the review by Lindén *et al.* [79]. Further information on the study of mucin glycomics, including identified O-linked glycan modifications characteristic of GI mucin and their biosynthetic pathways, can be obtained from glycobiology resources such as the Kyoto Encyclopedia of Genes and Genomes [80] and the Consortium for Functional Glycomics [81].

## 3. Adhesion

Most clinical studies of probiotic persistence and colonization show that probiotic organisms do not permanently colonize the GI tract and continue providing their hosts benefits only for brief periods after they have stopped being administered [82,83]. Little is known of what makes probiotic organisms so transient relative to commensals, so it is important to consider the factors influencing their capability to adhere and persist in the gut when studying and manipulating probiotic organisms. Bacteria adhere initially to GI surfaces by nonspecific physical interactions, such as steric and hydrophobic interactions, which result in reversible attachment. Several researchers have reported that there is a degree of correlation between hydrophobicity and adhesion to the hydrophobic mucosal surface [84,85,86,87]. However, other studies indicated that there was no correlation between cell surface hydrophobicity and adhesion to intestinal mucus [88,89]. In these studies, highly adhesive bacteria demonstrated fairly low surface hydrophobicities. This has suggested that cell surface hydrophobicity is not an accurate measure of adhesive potential. 

While adhesive characteristics of lactobacilli vary considerably among strains and species [90,91,92], many have large surface proteins with highly repetitive structures that are involved in mucus adhesion. Though specific mechanisms are not yet well understood, evidence suggests that carbohydrate-protein interactions play a key role in the adhesion of these proteins to mucin-bound oligosaccharides, especially considering numerous mucus-binding proteins contain regions homologous with binding domains of other known proteins such as lectins. The evolution of lectin-like adhesins in endosymbiotic bacteria may have been favored by the presence of multivalent substrates such as the mucins found in the GI tract. Affinities of lectins for multivalent glycoproteins can be 50-fold to 10^6^-fold greater than for individual glycan moieties [93]. Recently, a number of mucus-binding proteins have been isolated, some of which have been shown to display lectin-like interactions, and some of which may be conserved in numerous *Lactobacillus* species.

### 3.1. Mucus Binding Proteins

Several lactobacilli proteins have been shown to promote mucus adhesion (Table 2). The most studied example of mucus-targeting bacterial adhesins is the mucus-binding protein, MUB, produced by *L. reuteri* [82,94]. The MUB protein contains repeated functional domains, referred to by the authors as Mub domains, which are responsible for the protein’s adhesive properties. The Mub domain has since been designated a member of the MucBP domain family (Pfam PF06458). Numerous MUB homologues and MucBP domain containing proteins have been found, but almost exclusively in lactic acid bacteria and predominantly in lactobacilli found naturally in intestinal niches (Table 3). This suggests that MucBP domain containing proteins play an important role in establishing host-microbial interactions in the gut and promoted the evolution of the species as primarily GI organisms [93,95,96,97]. 

**Table 2:** Adhesion promoting proteins in *Lactobacillus* spp.

Protein	Info.	Species	References
MUB	Demonstrates binding to mucus *in vitro*	*L. reuteri*	[95]
MucBP Domain Containing Proteins	Contain MucBP domains, implicated in mucus adhesion	13 known *Lactobacillus* spp.	[98]
Pili	Pilin subunit SpaC binds to mucus *in vitro*	*L. johnsonii*, *L. rhamnosus*	[99,100,101]
32-Mmubp	Demonstrates binding to mucus *in vitro*	*L. fermentum*	[102]
SlpA	Knockouts show diminished adhesion to mucus *in vitro*	*L. acidophilus*	[103]
Msa	Demonstrates binding of mannose *in vitro*	*L. plantarum*	[104]
MapA	Demonstrates binding to mucus *in vitro*	*L. reuteri*	[105,106]
EF-Tu	Expression upregulated in the presence of mucus	*L. johnsonii*	[107,108,109,110,111]

**Table 3:** MucBP domain containing sequences in available *Lactobacillus* genomes.

Currently available whole genomes	Accession#	Gene	# of domains	Size
*Lactobacillus acidophilus* NCFM	Q5FKK8	*LBA0909*	1	508aa
	Q5FKA8	*LBA1017*	1	294aa
	Q5FKA7	*LBA1018*	1	346aa
	Q5FKA6	*LBA1019*	7	2650aa
	Q5FKA5	*LBA1020*	5	2310aa
	Q5FJS1	*LBA1218*	1	697aa
	Q5FJC2	*LBA1377*	2	1017aa
	Q5FJA7	*LBA1392*	17	4326aa
	Q5FJ43	*LBA1460*	2	339aa
	Q5FIQ0	*LBA1609*	2	643aa
	Q5FIL0	*LBA1652*	3	1174aa
	Q5FIF3	*LBA1709*	3	1208aa
*Lactobacillus brevis* ATCC 367	Q03U29	*LVIS_0122*	2	912aa
	Q03T21	*LVIS_0493*	3	1519aa
	Q03P66	*LVIS_1947*	1	1111aa
	Q03NB2	*LVIS_2262*	1	422aa
*Lactobacillus crispatus* ST1	D5H0E1	*LCRIS_00029*	3	1232aa
	D5H2Y1	*LCRIS_00919*	7	2935aa
	D5GXR1	*LCRIS_01123*	1	304aa
	D5GZ92	*LCRIS_01654*	2	3552aa
*Lactobacillus fermentum IFO* 3956	B2GFA4	*LAF_0157*	1	208aa
	B2GBH7	*LAF_0673*	2	1059aa
*Lactobacillus gasseri* ATCC 33323	Q047B3	*LGAS_0044*	4	873aa
	Q047B2	*LGAS_0045*	11	3692aa
	Q047B1	*LGAS_0046*	4	985aa
	Q046R7	*LGAS_0143*	6	2823aa
	Q045Q7	*LGAS_0410*	5	2457aa
	Q043P5	*LGAS_0939*	2	615aa
	Q043P2	*LGAS_0942*	10	2833aa
	Q043P0	*LGAS_0944*	1	524aa
	Q041C4	*LGAS_1655*	2	1425aa
	Q041B7	*LGAS_1663*	6	2449aa
	Q041A9	*LGAS_1671*	4	2552aa
	Q040V9	*LGAS_1725*	6	1993aa
*Lactobacillus helveticus* DPC 4571	A8YTV1	*lhv_0494*	1	155aa
	A8YTV2	*lhv_0495*	1	178aa
	A8YUX0	*lhv_0973*	1	278aa
	A8YUX3	*lhv_0979*	1	858aa
*Lactobacillus johnsonii* FI9785	D0R4C3	*FI9785_1070*	6	3401aa
	D0R5H6	*FI9785_1482*	5	1356aa
*Lactobacillus johnsonii* NCC 533	Q74LY7	*LJ_0046*	4	870aa
	Q74LY6	*LJ_0047*	6	2139aa
	Q74LY5	*LJ_0048*	4	983aa
	Q74L43	*LJ_0382*	4	3619aa
	Q74KU3	*LJ_0484*	4	4037aa
	Q74HP3	*LJ_0574*	5	1571aa
	Q74HU0	*LJ_0621*	5	2789aa
	Q74HW0	*LJ_0641*	3	1563aa
	Q74HA8	*LJ_1839*	7	1814aa
*Lactobacillus plantarum* JDM1	C6VP10	*JDM1_1038*	4	1082aa
	C6VQ03	*JDM1_1381*	6	2219aa
	C6VKM3	*JDM1_2438*	4	1345aa
	C6VL52	*JDM1_2491*	4	2037aa
	C6VL55	*JDM1_2494*	1	750aa
*Lactobacillus plantarum* WCFS1	Q88Y49	*lp_0946*	1	1189aa
	Q88XH5	*lp_1229*	3	1010aa
	Q88WI9	*lp_1643*	6	2219aa
	Q88UJ0	*lp_2486*	2	917aa
	Q88TB8	*lp_3059*	4	1356aa
	Q88T70	*lp_3114*	4	2032aa
	Q88T67	*lp_3117*	1	750aa
*Lactobacillus reuteri* DSM 20016	A5VKZ1	*Lreu_1258*	1	745aa
*Lactobacillus reuteri* JCM 1112	B2G8C6	*LAR_1192*	1	745aa
*Lactobacillus salivarius* CECT 5713	D8IM74	*HN6_01114*	4	785aa
*Lactobacillus salivarius* UCC118	Q1WSI9	*LSL_1335*	4	785aa

Data gathered from the Pfam [112] and Uniprot [113] databases; Databases contained no MucBP domain containing sequences in *Lactobacillus delbrueckii* subsp. *bulgaricus* strains ATCC 11842 and ATCC BAA-365, *Lactobacillus fermentum* CECT 5716, *Lactobacillus casei* strains Zhang, BL23, and ATCC334, *Lactobacillusplantarum* subsp. *plantarum* ST-III, *Lactobacillusrhamnosus* strains GG and Lc 705, and *Lactobacillussakei* subsp. *sakei* 23K.

MUB and most MucBP domain containing proteins exhibit characteristics typical of Gram-positive cell surface proteins; a *C*-terminal sortase recognition motif (LPXTG) for anchoring the protein to peptidoglycan, repeated functional domains and an *N*-terminal region signaling the protein for secretion [94,95].

Roos and Jonsson’s competitive adhesion study showed that the binding of MUB to mucus was inhibited by the glycoproteins fetuin and asialofetuin as well as fucose, suggesting that MUB interacts with specific muco-oligosaccharides [94]. The study also demonstrated equivalent adhesion to mucus from different hosts indicating that MUB binding has little to no host specificity regarding mucus components. The recent resolution of the crystal structure of a MucBP domain in MUB, dubbed Mub2 [92], and subsequent discovery of immunoglobulin binding, provides further evidence of a broad binding specificity. This suggests that binding specificities of MucBP domain containing proteins are dictated by multiple factors, not solely resulting from the presence of MucBP domains.

Fimbrial genes have been reported in *L. johnsonii* NCC533 [99], but the direct visualization of pili on *Lactobacillus* cells has only been shown for *L. rhamnosus* GG [100]. Fimbriae, also referred to as pili, are thin proteinaceous extensions from bacterial cells, predominantly in Gram-negative bacteria, that promote adhesion. In many pathogens, pili are virulence factors that promote attachment to the host [101]. Kankainen *et al.* [100] isolated a pilin subunit, SpaC, located within the pili structure and found at the pilus tip, which was concluded to be essential to the interaction of *L. rhamnosus* GG with host mucus. A mutant strain lacking *spaC* expression showed significantly reduced binding. While these genes are uncommon among lactobacilli, this study has shown for the first time that fimbrial interaction with mucus can mediate host adhesion in lactobacilli. 

SlpA, an S-layer protein in *L. acidophilus*,has been implicated in promoting adhesion directly to the GI surface, because *slpA* knockouts showed decreased adhesion capability [103]. However, this could possibly be due to disruption of other surface proteins. S-layer proteins and glycoproteins can form a latticed monolayer coating the surface of bacterial cells [114]. S-layer components can vary widely by species, but function to protect the cell from enzymatic damage, low pH, bacteriophages and phagocytosis. While S-layers are present in only some *Lactobacillus* species, they are beginning to be studied for their adhesive functions. A number of studies have begun associating S-layer proteins in probiotic bacteria with competitive exclusion of pathogens and pathogen adhesion to mucus [115,116,117].

Certain other surface proteins are implicated in contributing to adhesive properties of lactobacilli but are otherwise not well characterized or their importance to adhesive mechanisms is poorly defined. For instance, a 32 kDa protein associated with adhesion to porcine mucus in *L. fermentum*, named 32-Mmubp, was identified as a homologue of the substrate binding domains of the OpuAC ABC-transport protein family [102]. A mannose-specific adhesin protein (Msa, a MucBP domain containing protein) is responsible for the binding of mannose by *L. plantarum* [104]. While this was initially discovered as a protein responsible for agglutinating *Saccharomyces cerevisiae*,the presence of *L. plantarum* in many intestinal niches suggests that the MucBP domains of Msa may also play a role in adhesion to non-mannosylated muco-oligosaccharides as well. Elongation Factor Tu is a guanosine binding protein that is important in protein synthesis in the cytoplasm, but has been identified as a membrane associated protein as well [107,108]. More recently it has been found on the cell surfaces of many lactobacilli [109,110] and the demonstration of its upregulation in the presence of mucus suggests it may play a role in adhesion to the GI tract [111]. Mucus adhesion-promoting protein (MapA) is reported to mediate the binding of *L. reuteri* and *L. fermentum* to mucus [105,106]. Interestingly, it is also degraded into an antimicrobial peptide, which lends the host anti-pathogenic properties and provides an example of how large surface proteins may exhibit evolutionarily beneficial pleiotropic effects [118].

### 3.2. Factors that Influence Binding *in Vivo* and *in Vitro*

Numerous factors have been shown to influence binding of lactobacilli to mucus *in vitro*. Certain aspects of experimental design in particular should be reviewed when choosing or comparing methods to study adhesion *in vitro* because of the direct effects they have on adhesion. Time allotted for incubation of bacteria on immobilized mucus can have a significant influence on observed adhesion if microbial sedimentation occurs and the substrate is saturated at an artificial level [119]. 

Ramiah *et al.* [111] showed that growth conditions mimicking the GI environment have significant effects on the expression of several mucus adhesins *in vitro* in *L. plantarum*. *MapA* was upregulated 6-8-fold when incubated in the presence of mucin and up to 25-fold when exposed to physiological concentrations of pancreatin and bile compared to MRS grown controls. It was also found that *mapA* was significantly downregulated in the presence of cysteine, and suggested that cysteine is an effector molecule that represses transcription of *mapA*. *Mub* was expressed 80-140-fold more in the presence of mucin, but was suppressed 7-30-fold under normal gut physiological conditions containing bile and pancreatin. EF-Tu was expressed 33-100 times greater in media containing mucus, but was not affected by bile or pancreatin concentrations. This may connote interplay between different mechanisms regulating adhesin expression to adapt to particular environments.

The possible mechanisms whereby food components affect the adhesion of probiotic organisms *in vivo* have not been investigated thoroughly. Exposure to milk and milk fatty acids has been observed to reduce the adhesive properties of some probiotic lactobacilli [120,121] to human intestinal mucus *in vitro*, which may also be relevant *in vivo*. It is also hypothesized that entrapment in food matrices *in vivo*, resulting in binding to or steric hindrance of adhesins, can decrease adhesion of bacteria to intestinal surfaces [119].

All bacterial adhesion in the gut is also likely inhibited to some degree by competitive exclusion of access to binding sites by commensal organisms, but quantification of these effects have yet to be studied thoroughly.

## 4. *In Vitro* Models

Adhesion to the GI tract has been widely used as a criterion for the selection of probiotic lactobacilli [122]. It has generally been assumed that probiotic efficacy is enhanced by adhesion to the GI tract, which increases residence time *in vivo*. This extends the period during which probiotic organisms can exert beneficial effects, such as immune stimulation from contact with the intestinal tract [123,124]. However, it is difficult to link adhesion, specifically, with probiotic efficacy. Studies with isogenic strains containing adhesion factor knockouts [125] demonstrate decreased adhesion to the gut, however it is not known how such a knockout would alter probiotic efficacy. Adhesion has been demonstrated as an important factor in the displacement of pathogens by probiotic bacteria*in vitro* [126,127,128], but isolating the influence of adhesion*in vivo* is complicated by various confounded factors. The effects of probiotic bacteria stem not only from adhesion to the GI tract and competition for binding sites with pathogens, but from competition for nutrients as well as the production of exogenous antimicrobial and immune-stimulating compounds. Some studies do correlate adhesive capacity with immune response [129,130], but it is uncertain to what extent confounded factors may influence observed probiotic activity. Understanding the molecular mechanisms behind microbial adhesion in the gut could help determine the degree of probiotic functionality imparted by adhesion alone.

The validity of the experimental models used in the measurement of probiotic adhesion may, however, be difficult to interpret. No standard model for *in vitro* adhesion exists so findings vary widely not only between strains and species, but between models as well [131]. The *in vitro* model determines the nature of adhesion sites in the system; some cell culture models will emphasize the measurement of direct host-microbe cellular contact, whereas mucus-secreting cultures or immobilized mucus models will emphasize mucus and muco-oligosaccharide interactions more than other models. As summarized in Table 4, there are advantages and disadvantages to various types of *in vitro* adhesion models. It may therefore be important to study adhesion *in vitro* via different methods for a more thorough understanding of the interaction mechanisms most important to probiotic adhesion.

**Table 4:** Summary of *in vitro* adhesion models.

Model	Description	Advantages	Disadvantages	References
Immobilized mucus	Mucus preparations immobilized, usually in microtitre wells	Fast, isolates mucus-microbe interactions from other *in vivo* conditions	Difficult to separate mucus-specific from hydrophobic interactions	[91,131,132,133]
Cell culture	Polar monolayer of enterocytes resembling intestinal tissue	Provides conditions more similar to *in vivo* environment	Derived from cancer cells, could differ from healthy tissue. Not representative of cell-type ratios in mucosal epithelial tissues	
Caco-2/HT29	Caco-2 and HT29 carcinoma cell lines	Simple, well established in literature	Does not account for mucus presence	[134,135,136,137]
HT29-MTX/FU	HT29 culture treated with methotrexate or fluoruracil to secret mucus of different types	Accounts for presence of mucus	May not represent appropriate *MUC* gene expression	[138,139,140,141,142,143,144]
Co-cultures	Mixed culture of secreting and mucus-secreting cells	Better represents cell-type ratio of mucosal epithelial tissues	Little literature for use in adhesion studies	[145,146,147]
Whole tissue	Whole, intact or excised tissue	Provides *in vitro* conditions most similar to *in vivo* environment	Costly, difficult to obtain	
Resected tissue	Fragments of tissue excised from host	Mucus, epithelial tissue, and commensal organisms accounted for in model	Only small fragments at a time available from living hosts	[148,149]
Organ culture	Whole organs maintained *in vitro*	Better maintains the architecture of the tissue	Prohibitively expensive, may not function in same manner as *in vivo*	[150,151]

### 4.1. Mucus Adhesion Models

The simplest method to measure the adhesion of bacterial strains to mucus is by immobilizing commercially available mucin. Mucin is bound to microtitre well plates, bacterial culture is bound to the mucin and strains are compared thereafter in any number of methods, qualitatively or quantitatively [91,132,133]. The use of mucin alone in adhesion assays allows for the targeting of interactions between bacteria and particular mucins known to be expressed in a given host locations. It also isolates microbe-mucus interactions from other interactions, such as host cell-microbe interactions, which may or may not be desirable. One drawback of this model is the complication of microbial hydrophobic properties with mucus-binding properties. The comparison of hydrophobic binding interactions of bacteria to untreated polycarbonate wells with mucus binding interactions in treated wells in one study [131] showed that hydrophobic binding interactions are not easily separable from mucus binding interactions.

### 4.2. Cell-Culture Models

Cultures of human intestinal cell lines are often presumed to better represent the environment *in vivo* because of the presence of actual tissue. The availability of a simple *in vitro* intestinal tissue model, as in the Caco-2 cell line, has provided valuable insight into cellular interaction mechanisms that would have been much more difficult to obtain with more complex *in vitro* techniques or *in vivo*. Caco-2 and HT29 cells, the two most commonly used intestinal cell lines, can be grown in culture to form a homogeneous polar monolayer of mature enterocytes resembling the tissue of the small intestine [134]. These models were developed primarily for the study of absorption and permeability in the small intestine and are derived from intestinal carcinomas, so they may or may not be accurate models for adhesion to healthy colonic tissue [135]. Several studies have compared the extent of Caco-2 cell binding by potential probiotic bacteria to adhesion*in vivo* with mixed results [136,137]. Regardless, these cell lines do not take into account the omnipresent mucus layer found in the healthy intestinal tract. The HT29 cell line, however, can be treated with methotrexate (MTX) to differentiate the cells into mucin-secreting goblet cells [138,139]. The production of mucus by HT29-MTX cells increase adhesion of bacterial cells relative to Caco-2 or HT29 cells alone [140,141], further supporting the importance of the presence of mucus to bacterial adhesion. 

The HT29-MTX line is composed primarily of goblet cells, which incorporates a mucus layer into the model, but it still does not accurately represent the enterocyte/goblet cell ratio of the gut epithelial layer. In response to this drawback, Caco-2/HT29-MTX co-cultures have been developed [145,146,147]. Unfortunately, HT29-MTX differentiated goblet cells express *MUC5AC* and *MUC5B* at a much greater rate than *MUC2* [139], which could be a significant drawback when studying microbial adhesion to the colon, where MUC2 is prevalent. HT29 cells also differentiate in the presence of 5-fluorouracil (FU) to secrete MUC2 [142], and while this would emulate the colonic environment more closely, the HT29-FU model only seems to have been used in the study of pathogens thus far [139,140]. 

### 4.3. Whole Tissue Models

The disadvantage of many models is that they don’t take into account the presence of normal GI microbiota and the competitive exclusion that would take place *in vivo* between established commensal populations and exogenous microbes. For a more complete model of intestinal tissue *in vitro*, encompassing the mucus layer and epithelial tissue accurately, but also accounting for the presence of commensal microbiota, whole intestinal tissue fragments can be used [148,149]. Resected fragments of healthy colonic tissue may be difficult to obtain, but likely display characteristics closer to those probiotic bacteria would be expected to encounter *in vivo* than other models. Similarly, organ culture can be employed to maintain the viability and architecture of the tissue and has been used to assess adhesive properties of pathogens [150,151]. As of yet, it does not seem that organ culture has been used in the study of probiotic organisms; the expense of using organ culture is more easily justifiable with pathogenic organisms that could not otherwise be used in human models safely, unlike probiotic organisms.

## 5. Conclusion

As the field advances, discovery and selection of better probiotic organisms will become more sophisticated. Refinement of cell-culture techniques to better represent colonic environment could provide more accurate measures of adhesion, further aiding the selection of the best probiotic candidates for clinical trials. Printed glycan microarrays are beginning to be used to elaborate binding patterns of whole bacterial cells to different glycan structures [152]. Discoveries using similar techniques could promote the understanding of specific affinities for different binding proteins. Determining the structural characteristics and binding specificities of mucus-binding proteins improve our understanding of the mechanisms behind probiotic-host interactions. This could in turn lead to the development of better tools to select the most beneficial probiotic organisms, potentially opening the door for designer probiotics engineered or selected for desired host-responses. Likewise, a better understanding of host glycosyl legislation in the context of bacterial binding specificity could result in the development of probiotics targeted for specific hosts or host tissue. 

Regardless of what future advances may come, knowledge of the limitations within the study of bacterial adhesion, as in any field, should help in the interpretation of current discovery as well as with the planning of further research.
